# Stimulus Induced Rhythmic, Periodic, or Ictal Discharges (SIRPIDs) and its Association with Non-convulsive Status Epilepticus in Critically Ill Patients

**DOI:** 10.1177/15500594221095434

**Published:** 2022-04-27

**Authors:** Francesco Capecchi, Andrea di Giacopo, Emanuela Keller, Ian Mothershill, Lukas L. Imbach

**Affiliations:** 1 Department of Neurology, 27243 University Hospital and University of Zurich, Zurich, Switzerland; 2 Department of Neurology, Ente Ospedaliero Cantonale, 31033 Lugano, Switzerland; 3 Neurocritical Care Unit, Department of Neurosurgery and Institute of Intensive Care Medicine, University Hospital and University of Zurich, Zurich, Switzerland; 4 31033 Swiss Epilepsy Clinic, Klinik Lengg AG, Zurich, Switzerland

**Keywords:** SIRPIDs, status epilepticus, critically ill, recrutability theory, epileptic equivalent

## Abstract

Stimulus induced repetitive periodic or ictal discharges (SIRPIDs) are a commonly observed EEG pattern in critically ill patients. However, the epileptic significance of SIRPIDs remain unclear. We identified and reviewed 55 cases with SIRPIDs according to the ACNS criteria. SIRPIDs occurred after standardized painful stimuli during a standard 20-minute EEG. These cases were investigated regarding their relation to non-convulsive status epilepticus (NCSE) according to Salzburg Consensus Criteria and in-hospital mortality. In 37/55 patients (67.3%), SIRPIDs were associated with NCSE. In most patients (26/37 cases, 70.3%) with concurrent status epilepticus, SIRPIDs occurred after status epilepticus (on average 4.8 days later), but in 3/37 patients (8.1%) they were observed before a later status epilepticus. In four cases (4/37 cases, 10.8%), SIRPIDs appeared both before and after an episode of NCSE and in other four cases the two patterns coexisted in the same EEG. In 50% of the patients, status epilepticus was refractory, super-refractory or the patient died before its resolution. The overall mortality in the cohort was high at 58.2%. These findings corroborate the hypothesis that SIRPIDs might represent a state with increased epileptogenic potential, commonly co-occurring with NCSE. Furthermore, SIRPIDs are associated with therapy-refractory course of status epilepticus and high mortality.

## Introduction

Stimulus induced rhythmic, periodic, or ictal discharges (SIRPIDs) are a frequently unrecognized electroencephalographic finding in critically ill patients.^1–4^ SIRPIDs are defined by the American Clinical Neurophysiology Society as periodic or rhythmic electroencephalographic discharges “reproducibly brought up by an alerting stimulus”. The stimuli can be induced by acoustic, tactile and any other noxious or non-noxious stimulation and SIRDIPs can appear independently from clinical arousal.^5^ The EEG shows typically high amplitude self-limiting discharges that can resemble the electrographic pattern of a non-convulsive epileptic status.

The neuronal networks involved in the pathogenesis of SIRPIDs are unknown, but an underlying pathological cortico-subcortical dysregulation, associated with diffuse cortical hyper-excitability, has been suggested.^6,7^ Conversely, the influence of vigilance on epileptic activity in EEG recordings is a well-known phenomenon.^8,9^ In particular, phases of transition between different states of consciousness are susceptible to an enhancement of epileptic ictal or inter-ictal phenomena. For example, epileptic spikes are often associated with sleep wake transitional states (sleep onset and awakening). A similar mechanism might play a role in the emergence of SIRPIDs, considering its association to stimulation and change of vigilance states.

Since their first description in 2004, the significance of SIRPIDs and their epileptogenic potential has been debated.^10^ Many experts relate the pattern to the “ictal-interictal continuum”—**which reflects** its unclear significance and missing evidence for therapeutic implications.^11,12^ There is evidence for both epileptic and non-epileptic origin of the phenomenon. While few SPECT-based studies showed no reproducible change of cerebral blood-flow during SIRPIDs and thus argue against their ictal significance,^13,14^ other studies report a high prevalence of convulsive or non-convulsive status epilepticus (NCSE) over the course of the acute illness in patients with SIRPIDs.^15^ For instance, Braksick et al found a significant association between SIRPIDs and epileptic EEG findings, such as electrographic seizures, sporadic sharp-waves or periodic patterns in continuous EEG monitoring (cEEG).^15^ In the currently most comprehensive study on periodic and rhythmic discharges, Ruiz et al confirm an association between periodic patterns and NCSE, but the stimulus-responsivity was not predictive for a later to come ictal phase.^16^ Mostly observed in patients with deep alteration of consciousness or coma, SIRPIDs rarely lead to visible subtle clinical changes compared to baseline, but cases of responsiveness to benzodiazepines have been described.^17^ Most authors report a high mortality in **SIRPIDs cohorts**, whereas in a recent study, SIRPIDs did not seem to be independently associated with higher in-hospital mortality.^15,18,19^

In summary, these studies suggest that SIRPIDs are a pathological reaction to alerting stimuli, which appear in critically ill patients who frequently experience clinical or electroencephalographic epileptic activity over the course of the acute disease. However, the association of SIRPIDs with status epilepticus and its prognostic value in patients treated in an intensive care unit (ICU) remains unclear. Based on the clinical observation that many ICU patients with SIRPIDs also show EEG patterns of status epilepticus with similar frequency and morphology, we hypothesize that SIRPIDs might represent a state with increased epileptogenic potential. The primary aim of our retrospective, case controlled analysis is to assess the association between SIRPIDs and NCSE in our cohort. As a second endpoint, we assessed in-hospital mortality in the cohort showing SIRPIDs.

## Materials and Methods

**Figure 1.**
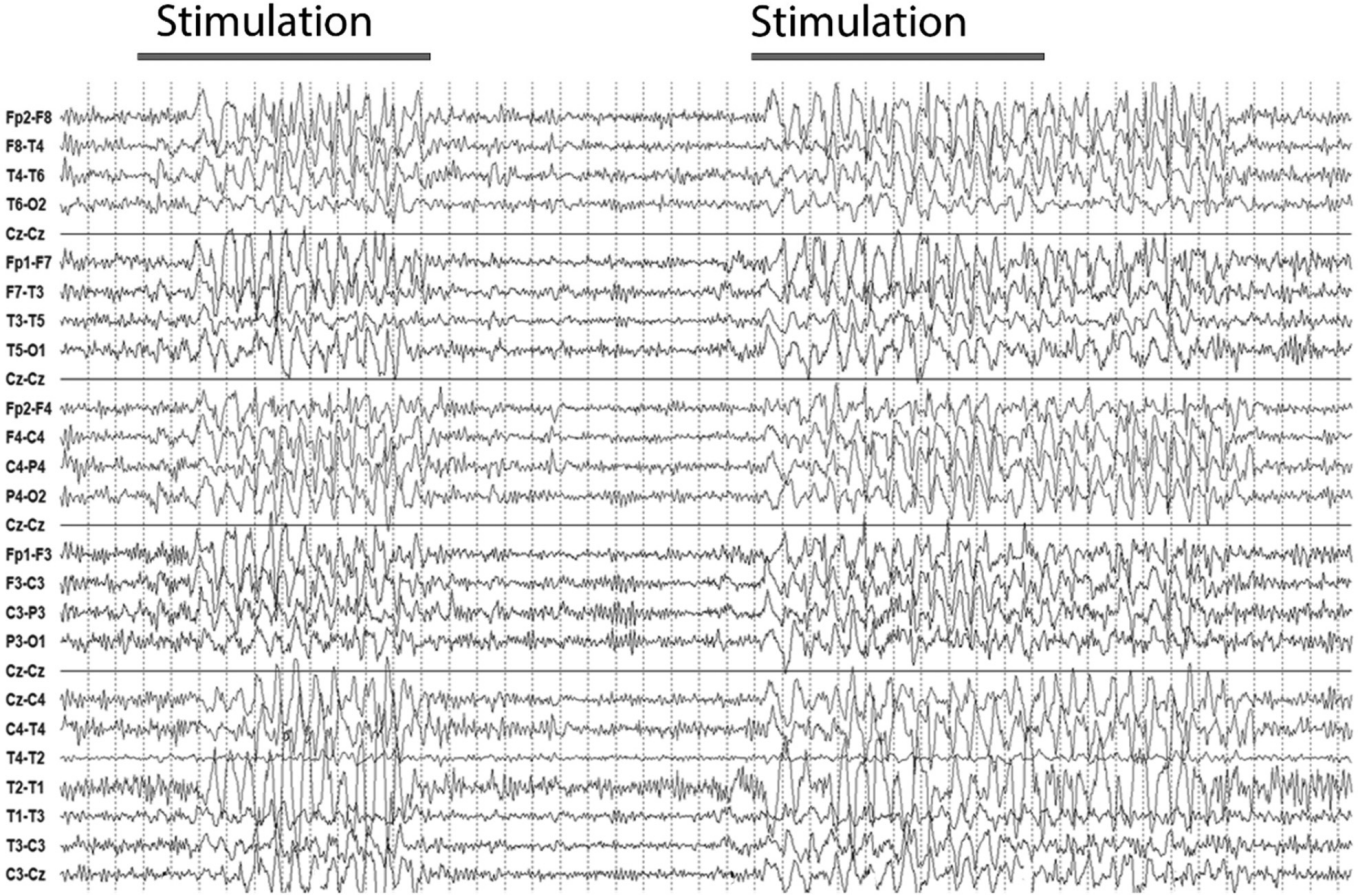
EEG over a time span of 50 seconds. Two episodes of SIRPIDs after stimulation on the right and left side. In particular, rhythmic and incompletely generalized sharp-and-slow- waves with a bifrontal maximum can be observed for respectively **9** and 17 seconds with a latency of up to one second after stimulation onset.

We screened our clinical database in the period from 1.1.2007 to 31.12.2020 for ICU patients who received at least two 20-min standard EEGs in our tertiary care center in a state of reduced vigilance. In total, we identified 67 patients with reproducible stimulus related EEG effects. These cases were then visually reviewed for electroencephalographic diagnosis of SIRPIDs according to criteria of the American Clinical Neurophysiological Society^5^ (Table 1: Patients’ Characteristics). A typical EEG with SIRPIDs is shown in Figure 1. Blinded review of these EEGs by two epileptologists (FC, LI) showed agreement in 55 patients (37 women, 67.3%, mean age 64.2 years). We excluded the 12 cases without agreement from further analysis. In the excluded EEGs, the time-locked appearance of the discharges after application of the stimulus could not be confirmed or a precise analysis of the EEG examination was not possible due to artifacts. Furthermore, we assessed the duration and the frequency of the SIRPIDs, as well as the electroencephalographic reactivity (changes of the EEG background from baseline after stimulation other than SIRPIDs), the dominant EEG background rhythm and the presence of other pathological discharges for all patients.

**Table 1.** Patients’ Characteristics.

Characteristics	Groups	Prevalence
Sex	Women	37/55 cases (67.3%)
Age	Mean age	64.2 years (median 69)
**Duration of the SIRPIDs**	**<10** **seconds**	**7/55 cases (12.7%)**
	**10–60** **seconds**	**31/55 cases (56.4%)**
	**>60** **seconds**	**17/55 cases (30.9%)**
Diagnosis	Cerebro-vascular disease	**32/55 cases (58.1%)**
	**Non-neurogenic shock**	**7/55 cases (12.7%)**
	**Unknown**	**5/55 cases (9.1%)**
	**Infection/Inflammation**	**4/55 cases (7.3%)**
	**CNS neoplasms**	**4/55 cases (7.3%)**
	**Genetic**	**2/55 cases (3.6%)**
	**Hepatic encephalopathy**	**1/55 cases (1.8%)**
Structural brain damage on CT/MRI	Present	**48/55 cases (87.3%)**
	Absent	**3/55 cases (5.5%)**
	No CT/MR performed	4/55 cases (7.3%)
Epilepsy specific findings	Prior epilepsy	6/55 cases (10.9%)
	Received AET	45/55 cases (81.8%)
	EEG Burst-suppression	11/55 cases (20%)
	**Spontaneous focal discharges**	**16/55 cases (29.1%)**
	**Spontaneous generalized discharges**	**3/55 cases (5.5%)**
	**Periodic/rhythmic focal delta waves**	**5/55 cases (9.1%)**
**Other EEG characteristics**		
**EEG reactivity**	**Present**	**47/55 cases (85.5%)**
	**Absent**	**8/55 cases (14.5%)**
**Posterior dominant rhythm**	**Alpha**	**0/55 cases**
	**Alpha/Theta**	**7/55 cases (12.7%)**
	**Theta**	**12/55 cases (21.8%)**
	**Theta/Delta**	**27/55 cases (49.1%)**
	**Delta**	**8/55 cases (14.5%)**
	**Burst-suppression**	**1/55 cases (1.8%)**

All EEG recordings except one were acquired in an ICU setting according to the 10/20 placement system (23 electrodes, recording time at least 20 minutes, sampling rate 200 Hz, off sedatives). One EEG showing SIRPIDs was recorded in the general neurological ward while the patient was still in a reduced vigilance state. As an alerting stimulus, we applied standardized painful stimulation of the fingernail as part of the standard EEG examination for all patients with reduced quantitative level of consciousness in our center. The application of the painful stimulus occurred after registration of at least ten minutes of EEG without stimulation. The stimulus was applied bilaterally with an interval of ca. 30 seconds between the two sides.

We investigated this cohort of **SIRPIDs patients** regarding the coexistence of NCSE (according to Salzburg consensus criteria)^20^ during their hospital stay, with a maximum of one month between the occurrence of SIRPIDs and the episode of NCSE. To this end, we reviewed all standard EEGs in this cohort during the hospital stay (313 EEGs, average 5.6 EEGs/case). NCSE was confirmed after 10 minutes of EEG registration. In the group with NCSE, we classified NCSE in therapy responsive, refractory or super-refractory depending on the responsivity to **Antiseizure drugs (ASDs)**. For this analysis we applied the responsivity criteria of status epilepticus and its definition proposed by Rossetti et al in 2011.^21^ Furthermore, we investigated the in-hospital mortality of the cohort and its relation to SIRPIDs with or without co-existence of NCSE.

As a control group, we selected 40 consecutive patients from a neurosurgical ICU between 2018 and 2020 who met following criteria: (1) Soporous or comatose state (2) ICU stay >3 days (3) at least two standard EEG performed during the hospital stay 4. Known cerebral pathology. The only exclusion criterion was the presence of SIRPIDs. In this group (22 women, 55%, mean age 63.7 years), we assessed the prevalence of status epilepticus and the in–hospital mortality, which we compared with those in the **SIRPIDs cohort**.

The local ethics committee approved the study, all patients or legal representatives gave informed consent. However, this consent did not include a provision stating that individual data can be made freely accessible.

## Results

We found episodes of NCSE in the majority of patients (37/55 cases, 67.3%) preceding or following the EEG diagnosis of SIRPIDs (Figure 1(A)).Four more patients who did not experience NCSE showed clinical seizures or electroencephalographic epileptic seizure patterns during their hospital stay (4/55 cases, 7.3%). In the control group, the prevalence of NCSE was 22.5% (9/40 cases) and the difference was statistically significant (odds ratio (OR) 7.08 [2.7889–17.9750], *p* < .0001).

**Figure 2.**
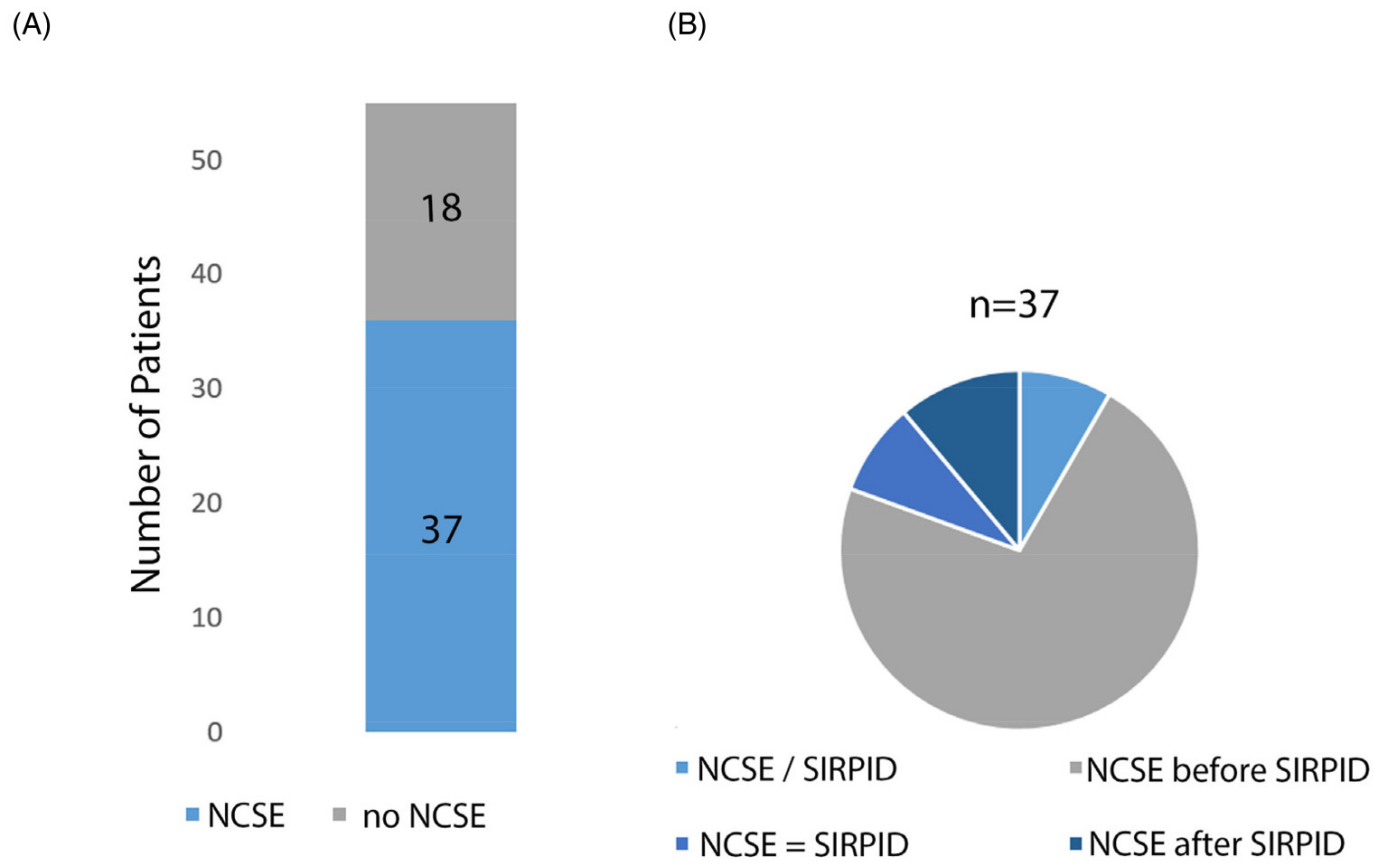
(A) prevalence of NCSE in our cohort of patients with SIRPIDs is represented (dark blue). (B) Patients are divided in four subgroups: NCSE occurring before SIRPIDs (**NCSE—SIRPIDs),** NCSE after SIRPIDs (**SIRPIDs—NCSE**), NCSE before and after SIRPIDs (**SIRPIDs—NCSE—SIRPIDs**) and NCSE in the same EEG as SIRPIDs (NCSE = SIRPID).

Next, we analyzed the temporal relationship between the occurrence of NCSE and SIRPIDs. In most **SIRPIDs cases**, status epilepticus occurred before the SIRPID pattern appeared (26/37 cases, 70.3%, average 4.8 ± 6.8 days before SIRPIDs, χ^2^ = 32.2667 *p* < .001). In three cases SIRPIDs were followed by NCSE (3/37 cases, 8.1%, average 2.6 ± 0.8 days), and in four cases (4/37 cases, 10.8%), electroencephalographic NCSE coexisted with SIRPIDs in the same EEG examination. In particular, in one case the EEG initially showed NCSE with continuous generalized rhythmic epileptic discharges, which disappeared shortly after administration of intravenous (IV) midazolam and briefly reappeared after painful stimulation. In another case, the EEG initially showed SIRPIDs; after multiple **stimulations** and faster background activity, spontaneous, high-frequency continuous discharges appeared and lasted until the end of the EEG examination. In the third case, we did not observe pathological discharges in the first minutes of the EEG, but two electroencephalographic seizures occurred time-locked to painful stimulation; afterwards, over the last ten minutes of the registration, eight clinical and electroencephalographic seizures followed without any external or visible internal stimulation. In the last case, the EEG showed a focal status epilepticus with periodic triphasic waves; after painful stimulation, the triphasic waves promptly became rhythmic and increased their frequency **before returning to baseline**. Finally, four patients showed EEG consistent with NCSE both before and after the occurrence of SIRPIDs (4/37 cases, 10.8%) (Figure 2(B)).

**Figure 3.**
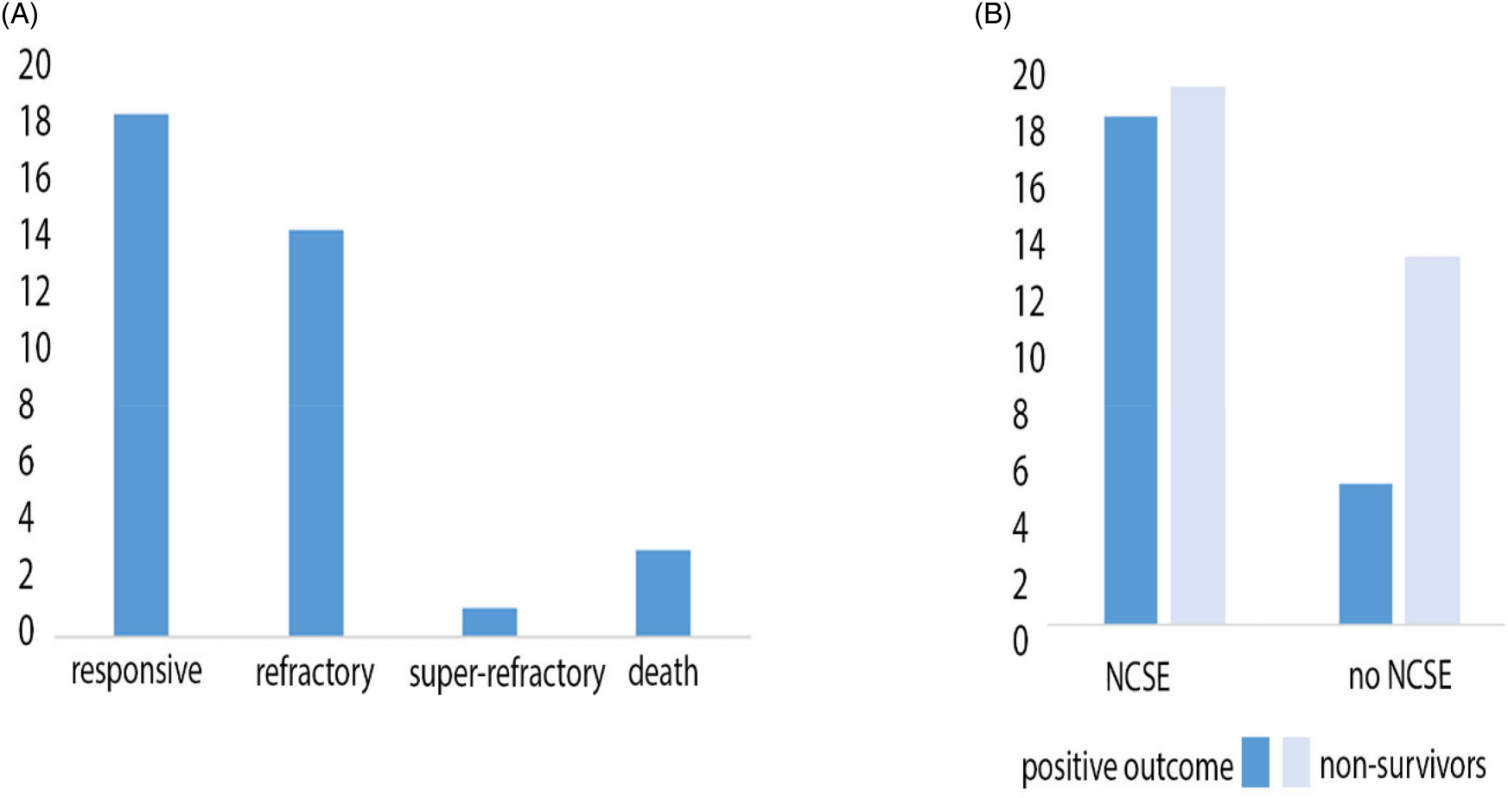
Treatment response and mortality in patients with and without NCSE. (A) **51.4% (19/37 cases)** of the cases of NCSE were responsive to standard treatment. In the other of the cases, NCSE was either refractory (**37.8%),** super-refractory (**2.7%)** or the patient died before resolution of the NCSE (**8.1%).** (B) Overall mortality was **58.2% (32/55** cases). The mortality in the group with NCSE was lower than in the group without NCSE (**51.4% vs 72.2%**).

**Figure 4.**
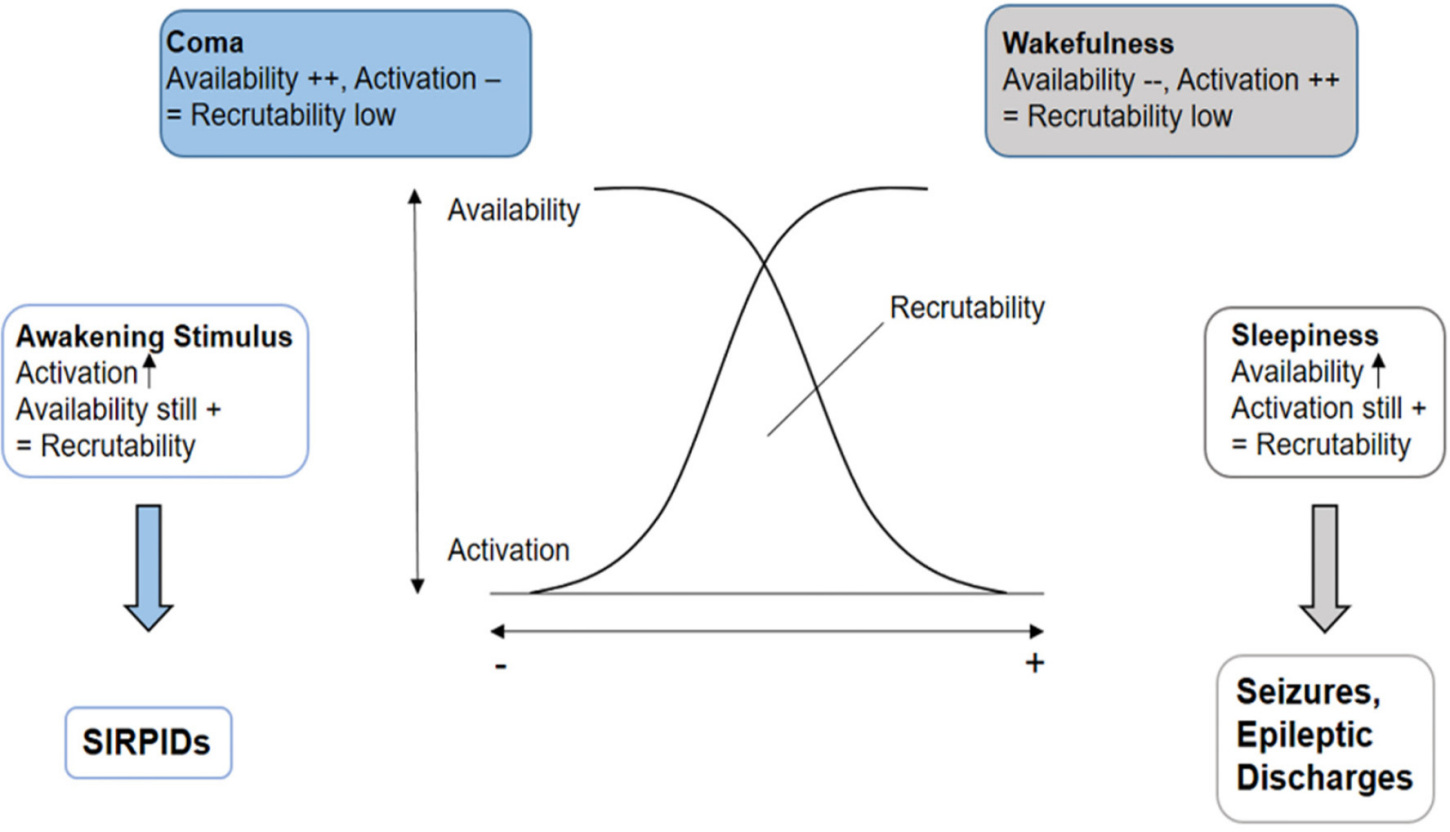
Modified graphical representation of the “recruitability theory” by Speckmann^22^ to explain the raised epileptogenicity of changes of vigilance states. On the right side of the graphic, neuronal recruitability increases (gray arrow) during the transition from wakefulness to light sleep due to the raise of the neuronal availability and persistence of the neuronal activation typical of wakefulness. In contrast, on the left side of the graphic, a raise of the recruitability can be observed (blue arrow) if neuronal activation increases, as deep coma is characterized by a very high availability of neuronal cells. We suggest that the activation caused by the awakening stimulus could lead to a transient elevation of the recruitability and, consequently, to SIRPIDs.

In the analysis of seizure responsivity based on the clinical medical records, we found that NCSE was **at least electroencephalographically** responsive to standard treatment in nineteen cases (19/37 cases, 51.4%). In fourteen cases, NCSE was refractory to initial treatment (14/37 cases, 37.8%) and in one case NCSE showed a super-refractory course (1/37 cases, 2.7%). **In many cases, a clinical improvement could be observed after resolution of the NCSE.** Three patients died before resolution of the NCSE (3/37 cases, 8.1%) (Figures 3(A) and 4). The overall mortality of our cohort was high at 58.2% (32/55 cases). In particular, mortality was higher in the group without NCSE (13/18 cases, 72.2%) **than** in patients who experienced NCSE during the hospitalization (19/37 cases, 51.4%), but the difference was not significant (χ^2^ = 2.16, *p* = .14) (Figure 3(B)). In the control group, mortality was significantly lower (15/40 cases, 37.5%; OR 2.32 [1.0063-5.3433], *p* < .0483).

**ASDs** were administered in 45/55 patients (81.8%) during the hospitalization. Thirty-five patients (35/55 cases, 63.6%) were already under **antiseizure medication** while SIRPIDs were observed. Ten patients (10/55 cases, 18.2%) were treated with IV thiopental/phenobarbital or high dose midazolam with burst suppression as a treatment for refractory status epilepticus. The mortality was lower (24/45 cases, 53.3%) in patients who received **ASDs** than in the group who did not receive **ASDs** (8/10 cases, 80%), but the difference was not significant.

IV midazolam was administered in sixteen cases during the EEG examination. All except one patient showed electroencephalographic responsivity of the SIRPIDs in terms of suppression of the rhythmic activity (15/16 cases, 93.8%), while no clinical response was observed.

Review of the available imaging data showed structural brain damage in all but four patients who received brain imaging (48/51 cases, 94.1%). In four cases, no neuroimaging was performed. Cerebro-vascular pathology was the most common cause of hospitalization in both **SIRPIDs patients** (32/55 cases, 58.2%) and the control group (27/40 cases, 67.5%). Posttraumatic cerebral hemorrhages were also included in this subgroup. In seven cases (7/55, 12.7%), SIRPIDs occurred in patients with severe cardiogenic, hemorrhagic or septic encephalopathy, including three patients after out-of-hospital reanimation. Cerebral neoplasms and inflammatory or infectious central nervous system disease were responsible for four cases each (4/55 cases, 7.3%). Two patients had a genetic epilepsy (2/55 cases, 3.6%) and one patient had hepatic encephalopathy. In five cases, the diagnosis was unknown (Table 1). Six patients were previously diagnosed with epilepsy (6/55 cases, 10.9%).

The mean duration of SIRPIDs was 85 seconds ± 118 seconds, with great variability from a minimum of five seconds to a maximum of ten minutes. In only seven patients, SIRPIDs lasted less than 10 seconds (7/55 cases, 12.7%). In most cases, the duration was between ten and sixty seconds (31/55 cases, 56.4%), while in seventeen cases SIRPIDs lasted more than one minute (17/55 cases, 30.9%). Moreover, we compared the mean duration of SIRPIDs in patients with or without status epilepticus (83.5 ± 126.9 seconds vs 88.2 ± 103.1 seconds, t = 0-137, *p* = .89) and between non-survivors and survivors (92.4 ± 112.6 seconds vs 74.9 ± 124.6 seconds, t = −0.535, *p* = .59). Direct pairwise statistical comparison did not reveal any significant difference between the subgroups. The frequency of rhythmic discharges in SIRPIDs was between 1/s and 2/s in all but one case, where we observed particularly slow periodic discharges with a frequency of 0.5/s.

Most patients showed a preserved EEG background reactivity to painful stimuli other than SIRPIDs (47/55 cases, 85.5%). There was no significant difference between patients with (32/37 cases, 86.5%) or without NCSE (15/18 cases, 83.3%) or patients who died (26/32 cases, 81.2%) or not (21/23 cases, 91.3%).

We classified the posterior dominant rhythm in alpha, alpha/theta, theta, theta/delta, delta or burst-suppression. In most cases we observed a mixed theta/delta activity (27/55 cases, 49.1%), corresponding to a moderate slowing of the background activity. In about one third of the patients, we found mild background slowing with isolated theta (12/55 cases, 21.8%) or admixture of alpha/theta activity (7/55 cases, 12.7%). A severe, delta slowing was present in eight patients (8/55 cases, 14.5%) and the last patient had a burst-suppression pattern. We found no significant differences between the subgroups. Finally, we looked for the presence of spontaneous focal or generalized, ictal or interictal discharges or periodic/rhythmic patterns. Focal discharges were the most frequent finding (16/55 cases, 29.1%), while we found generalized triphasic waves only in three patients (3/55 cases, 5.5%). In five more patients we observed spontaneous focal periodic or rhythmic delta waves, mostly bifrontal (5/55 cases, 9.1%). Again, there was no significant difference regarding our primary and secondary outcome variables among the subgroups.

## Discussion

In this study, we examined the prevalence of NCSE in an ICU cohort of **SIRPIDs patients** and its temporal association with the diagnosis of SIRPIDs. As the main finding, we found a very high prevalence of NCSE (67.3%) in this cohort and a seven-fold increased risk of developing NCSE as compared to the control group. Furthermore, SIRPIDs appeared in close temporal association, few days before or after the episodes of NCSE. In the majority of the cases, they appeared shortly after the episodes of NCSE, while **in seven** cases NCSE followed the diagnosis of SIRPIDs. Finally, we provide further evidence that SIRPIDs in critically ill patients are associated with high mortality. The duration of the SIRPIDs, as well as other EEG findings like EEG reactivity, posterior dominant rhythm and focal or generalized discharges, did not have a relevant influence on the risk of the developing NCSE or on the mortality in our cohort.

Up to **now**, the clinical impact of SIRPIDs, their epileptological relevance and its treatment strategy remain uncertain. However, SIRPIDs may represent a suppressed epileptic phenomenon. This conclusion is based on several findings: First, the co-occurrence of NCSE and SIRPIDs in the majority of cases suggests a direct association between these electroencephalographic patterns. Moreover, the fact that SIRPIDs are mainly observed after NCSE under sedative treatment supports the hypothesis that SIRPIDs may represent partly suppressed rhythmic epileptic activity that reemerges upon stimulation. Finally, the responsivity of SIRPIDs to benzodiazepines in many cases and the nearly exclusive occurrence in patients with known structural cerebral damage are further clinical parallels of SIRPIDs and NCSE.

From a neurophysiological point of view, we support the interpretation that SIRPIDs might be an equivalent of NCSE in deep unconscious patients due to brainstem and thalamic dysfunction or pharmacological intervention, as proposed by Hirsch et al.^5^ A possible explanation for this is based on the increased epileptogenicity during changes of vigilance states, according to the “recruitability model” proposed by Speckmann in 1986.^22^ In this model, neuronal recruitability (= the probability of a neuronal cell or a neuronal circuit to be recruited by an epileptic focus) depends on the state of activation of cortical neurons and on their availability, which reflects the number of neuronal cells which could potentially be recruited by an epileptic focus at a certain time. On the one side of the spectrum, active wakefulness is characterized by high activation of cortical neuronal cells, but low availability, as most neurons are already processing the constantly incoming afferent information. The opposite happens in coma, where cortical activation is at its lowest, which leads to low recruitability despite the high number of available neuronal cells. Thus, both conditions are not favorable for the **intrusion** and spreading of epileptic activity. During the transition phase from wakefulness to light sleep, more neuronal cells become suddenly available, while an elevated activation level persists, leading to increased recruitability and, consequently epileptogenicity.^7,8^ We suggest an analogous mechanism to underlie the occurrence of SIRPIDs in comatose patients with focal or generalized cortical hyper-excitability, whose level of unconsciousness is too deep (and neuronal activation too low) to allow spontaneous seizure activity. The change of vigilance state in this case is triggered by the painful stimulus, which leads to an **nonspecific** arousal with desynchronization of the cortical electrical activity through specific and unspecific thalamic projections mainly to the primary somato-sensory cortex and the limbic system. The result of this transient raise of neuronal activation within a background of augmented availability typical of the deep coma leads to a significant increase of the neuronal recruitability and epileptogenicity.

This mechanism could also explain the higher mortality in cases who did not experience status epilepticus. In particular, the deeper level of unconsciousness of these patients, which does not allow the **appearance** of spontaneous epileptic activity during the course of the disease due to lack of neuronal activation, could be expression of more severe cerebral damage and poor prognosis. Alternatively, one could argue that patients with documented NCSE were more likely to be treated with **ASDs**. Now, if we assume that SIRPIDs represent a state of higher epileptogenicity, patients without concurrent NCSE might therefore not have received the adequate **antiseizure medication**. In line with this, the high prevalence of patients with structural brain damage in our cohort suggests that patients with SIRPIDs, who are able to recover from coma, are probably at higher risk of developing NCSE, if the brain pathology causing the cortical hyper-excitability is not resolved.

We recognize that this study has several limitations, such as retrospective, non-standardized patients’ selection based on EEG pattern or different underlying brain pathologies, which probably had an influence on the mortality data in our cohort. Thus, the retrospective nature of the study does not allow to establish a causal relation between SIRPIDs and NCSE. Furthermore, the lack of a standardized treatment protocol in subjects with or without NCSE does not allow us to draw conclusions on the optimal therapeutic approach in patients with SIRPIDs. It remains an open question, whether the pragmatic initiation of an **antiseizure medication** can be considered on an individual basis in treatment-naïve patients with SIRPIDs. Future analysis should clarify, if the initiation of an antiepileptic first-line drug in patients with SIRPIDs could reduce the imminent risk of NCSE over the course of the disease.
